# Genomic testing for *RET* in the clinic: UK and global perspective

**DOI:** 10.1530/ERC-24-0230

**Published:** 2025-04-15

**Authors:** Louise Izatt

**Affiliations:** ^1^ Consultant in Clinical Genetics, Guy’s and St Thomas’ NHS Foundation Trust, Guy’s Hospital, London, United Kingdom; ^2^ Honorary Senior Lecturer, Department of Medical and Molecular Genetics, Guy’s Campus, King’s College London, London, United Kingdom

**Keywords:** *RET*, genomic test, pathogenic variant, MEN2, MTC

## Abstract

**Graphical abstract:**

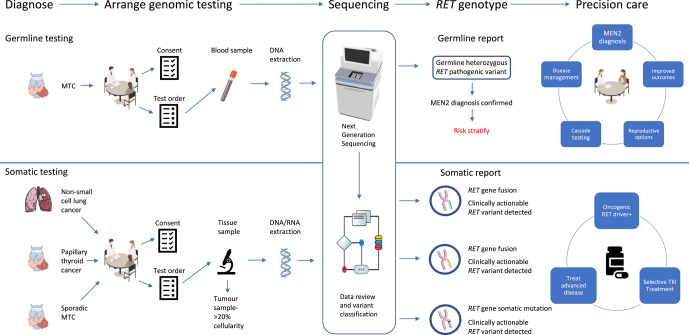

**Abstract:**

*RET* is a key oncogene in neuroendocrine cancer. Pathogenic germline variants lead to multiple different phenotypes, including multiple endocrine neoplasia type 2, medullary thyroid cancer (MTC), Hirschsprung disease and kidney malformations. Pathogenic somatic variants are also associated with MTC, and *RET* rearrangements are observed in papillary thyroid cancer, non-small cell lung cancer and pan-cancer syndromes. Testing for both germline and somatic variants is now feasible in everyday clinical practice, and their identification has important clinical consequences, both for affected individuals and their families. This mini-review will discuss current germline and somatic testing strategies in the UK and worldwide, as well as reporting and test outcomes (including variants of uncertain significance or incidental findings). It will explore actions following identification of a pathogenic germline variant, including predictive, reproductive and childhood testing, and somatic testing of *RET* variants in solid tumours informing personalised cancer treatment. Finally, it will discuss the challenge of delivering rapid and equitable access to genomic testing to ensure that all individuals can benefit promptly and appropriately to improve clinical outcomes.

## Introduction

Genomic testing has expanded greatly in the last decade, shifting from limited access within clinical genetics services into mainstream care. Specialists from multiple areas now routinely test for germline and/or somatic variants early in their patients’ pathways to determine whether there is a heritable cause and to inform mutation-specific clinical care (Eggermann *et al.* 2020). This transformation was possible due to technical advances in DNA sequencing and computational biology, reduced sequencing costs, more efficient variant interpretation, improved test turnaround times and upskilling in workforce understanding and utilisation – all of which have contributed towards earlier genomic diagnoses with prognostic, therapeutic and management implications for personalised care.

In 2019, the UK leveraged the 100,000 Genomes Project to commission a national Genomic Medicine Service (GMS) (Brittain *et al.* 2017), which now provides the entire English population with access to genomic tests for inherited rare diseases and cancer (similar services exist in Scotland, Wales and Northern Ireland). All clinicians can access the National Genomic Test Directory (NGTD) (https://www.england.nhs.uk/publication/national-genomic-test-directories/; accessed 26/08/2024), which lists available genomic tests (from whole genome sequencing (WGS) to single-gene testing), describes the technology used to deliver testing, and details patient eligibility criteria. Somatic testing is available to aid diagnosis and management of solid tumours and give prognostic information, identifying oncogenic drivers for targeted therapies. Testing is delivered through core and specialised arrangements within seven networked Genomic Laboratory Hubs, working to common national standards, specifications and ISO accreditation (ISO 151989:2012). All genomic tests are funded by NHS England at no direct cost to the patient or health provider. Globally, access to *RET* genomic testing is performed in multiple laboratories (https://www.orpha.net/en/diagnostic-tests/diagnostics/; accessed 12/01/2025) or (https://www.ncbi.nlm.nih.gov/gtr/; accessed 12/01/2025). Access to genomic testing, the extent of testing, techniques employed and reimbursement of costs will be country and healthcare system-dependent, guided by national guidelines, for example, https://www.nccn.org/professionals/physician_gls/pdf/thyroid.pdf; accessed 12/01/2025. Provided criteria for testing are fulfilled (for example, a patient presenting with medullary thyroid cancer (MTC)), insurers will often cover the cost of both germline and somatic *RET* testing in the USA and Europe.

## *RET*: a key endocrine oncogene

### Gene structure

**Figure 1:**
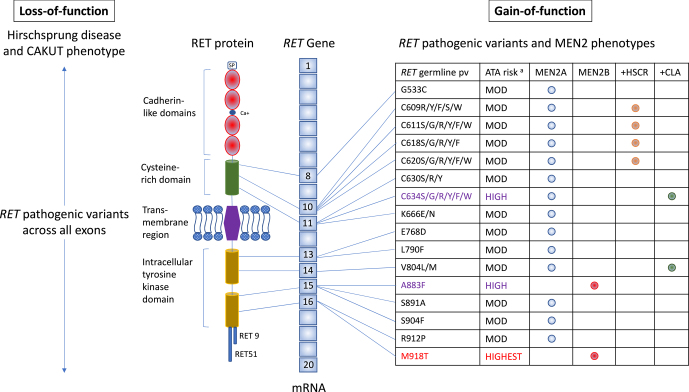
Schematic representation of the *RET* gene, protein and phenotypes associated with common *RET* germline pathogenic variants. Representation of the *RET* gene (middle), RET protein (left) and phenotypes associated with LoF or GoF germline pathogenic variants (pv) (listed on right). The *RET* gene is located on 10q11.2, containing 20 exons. It encodes a transmembrane receptor which has a signal peptide, four cadherin-like domains and a cysteine-rich domain sited extracellularly. The intracellular domain contains a tyrosine kinase domain. The two main alternatively spliced isoforms of RET protein are RET9 and RET51. Hirschsprung disease (HSCR) and congenital anomalies of the kidney and urinary tract (CAKUT) occur with LoF pv across the entire coding sequence of the *RET* gene. In MEN2, GoF pv, listed as amino acid changes, are clustered in specific exons 8, 10, 11 and 13–16, as shown. ^a^The most frequent *RET* pvs and MEN2 phenotypes are shown in black, purple and red to represent the moderate (MOD), high and highest risk of MTC according to the Revised ATA 2015 guidelines (Wells *et al.* 2015). The MEN2 phenotypes associated with various common *RET* germline pvs are listed in the table on the right. +HSCR, +CLA, cutaneous lichen amyloidosis. Data from Waguespack *et al.* (2011).

The gene *RET* (rearranged during transfection) was identified in 1985 as an oncogene by its ability to transform NIH3T3 cells by DNA rearrangement (Takahashi *et al.* 1985). *RET* encodes a transmembrane RET that regulates cell migration, proliferation, survival and differentiation (Fig. 1). Ret also has a key role in the development of the renal tract, enteric nervous system and other neuroendocrine pathways; it is therefore unsurprising that *RET* germline mutations result in clinical phenotypes affecting these systems (Mulligan 2014).

### *RET* pathogenic variants

Dysregulation of *RET* via loss-of-function (LoF) or gain-of-function (GoF) mechanisms results in multiple autosomal dominant conditions.

Germline LoF pathogenic variants can cause Hirschsprung disease (Edery *et al.* 1994) or kidney malformations (Pini Prato *et al.* 2021). Germline GoF pathogenic variants cause MEN2, where the hallmark of the disease is MTC (Mulligan 2014). MEN2-associated variants cluster in exons 8, 10, 11 and 13–16 and are mostly single-nucleotide variants, although deletions, duplications, insertions, homozygotes or tandem variants in a *cis* configuration also occur (Fig. 1) (Elisei *et al.* 2019, Salvatore *et al.* 2021).

Considering somatic variants, *RET* GoF pathogenic variants are found in approximately half of sporadic MTC cases (Ciampi *et al.* 2019), and activating *RET* chromosomal rearrangements or gene fusions constitute oncogenic drivers in multiple solid tumours, including 10–20% papillary thyroid cancers (PTCs) (Agrawal *et al.* 2014) and 1–2% of NSCLC (Takeuchi *et al.* 2012). Somatic or germline *RET* alterations are druggable targets, which enables personalised cancer treatments (Filetti *et al.* 2022).

## Clinical disease associated with germline *RET* pathogenic variants

### MTC and MEN2

**Figure 2:**
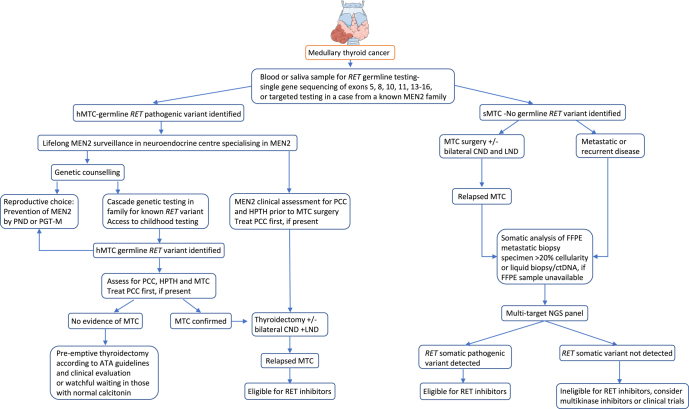
Recommended *RET* genomic testing for patients presenting with MTC and personalised care. 75% of MTC cases are sporadic (sMTC) and 25% are syndromic, described as hereditary (hMTC). Testing of specific exons of *RET* exons 5, 8, 10, 11 and 13–16 is recommended, as the known GoF pathogenic variants leading to hMTC or sMTC are concentrated in these exons. MEN2, multiple endocrine neoplasia type 2; PCC, phaeochromocytoma; HPTH, hyperparathyroidism; PND, prenatal diagnosis; PGT-M, pre-implantation genetic testing for monogenic disorders; ATA guidelines, revised American Thyroid Association Guidelines (Wells *et al.* 2015); bilateral CND and LND, bilateral central neck dissection and lymph node dissection; NGS, next-generation sequencing. FFPE (formaldehyde-fixed-paraffin-embedded tissue) is required for somatic analysis, or ctDNA (circulating tumour DNA). *RET* testing algorithm data adapted from Belli *et al.* (2021).

MTC (OMIM #155240) arises from the parafollicular C-cells of the thyroid gland. MTC is rare, accounting for ∼2–3% of thyroid cancers (TCs), with 107 cases reported in England in 2021 (National Disease Registration Service). 75% of MTC cases are sporadic; however, 25% are syndromic, occurring in patients with germline *RET* variants and MEN2 (Fig. 2).

Ninety-five percent of MEN2 cases have MEN2A (OMIM #171400), characterised by developing MTC with a variable risk of phaeochromocytoma and hyperparathyroidism. MTC is usually the presenting tumour in MEN2A, but in 30% of cases, phaeochromocytoma develops first, whereas hyperparathyroidism is an uncommon presentation (Rodriguez *et al.* 2008, Larsen *et al.* 2020). Some MEN2A cases additionally present with Hirschsprung disease (codons 609, 611, 618 or 620) or cutaneous lichen amyloidosis (codons 634 or 804) (Fig. 1) (Verga *et al.* 2003, Coyle *et al.* 2014).

MEN2B (OMIM #162300) (5% of MEN2 cases) arises in individuals with a pathogenic variant M918T or, infrequently, A883F (Mathiesen *et al.* 2017). M918T patients present with early-onset, aggressive MTC and 50% develop phaeochromocytoma, but hyperparathyroidism is not described. Other pathognomonic non-endocrine features include intestinal ganglioneuromatosis, marfanoid habitus, mucosal neuromas of the lips and tongue, and corneal nerve thickening (Castinetti *et al.* 2018). Furthermore, >90% M918T cases are *de novo* (Brauckhoff *et al.* 2014).

Strong genotype–phenotype correlations are described in MEN2 (Eng *et al.* 1996). The American Thyroid Association (ATA) classifies *RET* variants into three risk levels for the age of onset/aggressiveness of MTC (highest, high and moderate) (Wells *et al.* 2015). Patients with *RET* M918T represent the highest risk category, with the earliest age of MTC onset, whereas C634X or A883F variants are categorised as high risk. In contrast, all other MEN2A variants are categorised as moderate risk (Machens & Dralle 2024).

Up to 7% cases of apparently sporadic isolated MTC have germline MEN2A *RET* pathogenic variants, predominantly non-cysteine variants, e.g., V804M, with a moderate MTC risk (Romei *et al.* 2011), whereas ∼55% of sporadic MTC cases have a somatic *RET* variant (Fig. 2) (Ciampi *et al.* 2019).

### Hirschsprung disease

Hirschsprung disease (OMIM#142623) is characterised by the congenital absence of myenteric and submucosal plexuses in the distal colon and rectum. Isolated Hirschsprung disease is most frequently caused by LoF *RET* variants (Fig. 1). Hirschsprung disease can also occur in other genetic syndromes (Karim *et al.* 2021), including MEN2A, where co-segregation occurs in individuals with *RET* codon 609, 611, 618 and 620 pathogenic variants (Arighi *et al.* 2004, Moore & Zaahl 2012).

### CAKUT abnormalities

LoF *RET* variants are described in congenital absence of the kidney and urinary tract (CAKUT) cases. Some patients have both CAKUT and Hirschsprung disease (Fig. 1) (Davis *et al.* 2014).

## Testing approach for *RET* (MEN2) germline variants

### Who to test

*RET* genetic testing is recommended for all patients suspected of having an inherited *RET* pathogenic variant to inform medical management and targeted treatment in MEN2 and to determine familial risk. Early thyroidectomy, before MTC develops, is recommended in MEN2 families to reduce MTC mortality (Wells *et al.* 2015).

Pre-test evaluation of the affected patient includes personal and family history, clinical phenotyping and collation of biochemical, histological, imaging and prior personal or familial genomic test results to confirm eligibility and inform the genomic testing strategy (Izatt *et al.* 2022). Table 1 lists eligibility criteria for *RET* germline testing.

**Table 1:** Eligibility criteria for *RET* germline testing.

Patients who are eligible for *RET* germline testing include*	
1	An individual diagnosed with MTC (any age)
2	An individual with ≥2 MEN2-related endocrine abnormalities (any age)
3	An individual with ≥1 MEN2-related endocrine abnormality and a first-degree relative with ≥1 MEN2-related endocrine abnormality (any age). MEN2 abnormalities include MTC, phaeochromocytoma/paraganglioma, parathyroid adenoma/hyperplasia or Hirschsprung disease
4	An individual with Hirschsprung disease (any age)
5	An individual with a *RET* pathogenic germline variant identified in a close relative
6	An individual with a potential *RET* pathogenic germline variant identified on somatic testing^†^

* The clinical indication for genomic testing and the eligibility for accessing the test are listed in the National Genomic Test Directory (NGTD) for rare and inherited diseases (https://www.england.nhs.uk/wp-content/uploads/2024/07/national-genomic-test-directory-rare-and-inherited-disease-eligibility-criteria-v7.pdf; accessed 26/08/2024).

^†^ A potential *RET* germline pathogenic variant might be present if insertions/deletions <50 bp >20%, or if a single nucleotide variant >30% variant allele frequency is detected on somatic tumour testing, regardless of tumour type.

Informed written consent before testing should be obtained. This will include discussion of the benefits and limitations of testing (including the possibility of uncertain or unexpected results), the implications of results for the patient’s own care and for their family (including cascade testing and reproductive decision-making), storage of DNA and sequencing data, and recording of the result in the individual’s medical record.

### Test choice

In individuals with MTC, MEN2 or Hirschsprung disease, only *RET* gene testing is recommended (Rare and inherited disease eligibility criteria; https://www.england.nhs.uk/publication/national-genomic-test-directories/; version 7:2024; accessed 26/08/2024). However, in other laboratories, a next-generation sequencing (NGS) panel of endocrine genes, including *RET*, is employed because it is more efficient and cost-effective. In the UK, this is the testing strategy for individuals with phaeochromocytoma (<60 years), bilateral phaeochromocytoma or hyperparathyroidism (<50 years), which includes *RET* in the multigene NGS panel. The probability of detecting *RET* variants by clinical presentation and genetic test choice is detailed in Table 2. As the criteria for testing, choice of genes on an endocrine panel and process for testing will vary in different healthcare settings, the clinician is advised to confirm local provision.

**Table 2:** Probability of detecting a *RET* pathogenic variant by clinical indication.

Clinical presentation*	Probability of detecting a germline *RET* pathogenic variant	Test required^†^
MTC, any age	25% (Romei *et al.* 2011)	Sequencing of selected *RET* exons 5, 8, 10, 11, 13, 14, 15, 16^‡^
MEN2A clinical diagnosis (with a confirmed family history)	98% (Elisei *et al.* 2019)	Sequencing of selected *RET* exons 5, 8, 10, 11, 13, 14, 15, 16^‡^
MEN2B clinical diagnosis	100% (Elisei *et al.* 2019)	Sequencing of selected *RET* exons 5, 8, 10, 11, 13, 14, 15, 16^‡^
Familial MTC	98% (Elisei *et al.* 2019)	Sequencing of selected *RET* exons 5, 8, 10, 11, 13, 14, 15, 16^‡^
Sporadic MTC	7% (Romei *et al.* 2011)	Sequencing of selected *RET* exons 5, 8, 10, 11, 13, 14, 15, 16^‡^
Sporadic phaeochromocytoma without syndromic features	3.6% (Fussey *et al.* 2021)	Sequencing of a small panel of 11 phaeochromocytoma and paraganglioma genes including selected *RET *exons^‡^
Bilateral phaeochromocytoma	50% (Neumann *et al.* 2019)	Sequencing of a small panel of 11 phaeochromocytoma and paraganglioma genes including selected *RET *exons^‡^
Primary hyperparathyroidism (HPTH)	<1% (Larsen *et al.* 2020)	Sequencing of a small panel of eight FHH (familial hypocalciuric hypercalcaemia) and HPTH genes, including selected *RET *exons^‡^
Isolated Hirschsprung disease-sporadic	Up to 20% (Attié *et al.* 1995)	Sequence entire *RET* gene (20 exons)
Familial Hirschsprung disease	50% (Attié *et al.* 1995)	Sequence entire *RET* gene (20 exons)
Patient with a first degree relative with a *RET* pathogenic germline variant	Up to 50%	*RET* variant specific sequence test, targeting the known familial pathogenic variant
Patient with a potential *RET* pathogenic germline variant identified on somatic testing	47.8% (Kuzbari *et al.* 2023)	*RET* variant specific sequence test, targeting the potential *RET* variant

* The clinical indication for genomic testing and the eligibility for accessing the test are listed in the National Genomic Test Directory (NGTD) for rare and inherited diseases (https://www.england.nhs.uk/publication/national-genomic-test-directories/; accessed 26/08/2024).

^†^ The type of test required is listed on the NGTD. The genes in the rare disease panel are listed on PanelApp (https://nhsgms-panelapp.genomicsengland.co.uk/; accessed 26/08/2024).

^‡^ The list of selected *RET* exons is as recommended in the 2012 European Thyroid Guidelines (Elisei *et al*. 2012). However, the inclusion of exon 5 is under review by endocrine specialist genomic testing laboratories in England.

### Genomic analysis and reporting

Identified variants are classified using American College of Medical Genetics (ACMG) and Association of Clinical Genomic Sciences guidelines (Richards *et al.* 2015, Loong *et al.* 2022). In addition, for MEN2-associated *RET* variants, modified ACMG guidelines are available (Margraf *et al.* 2022). These frameworks use multiple strands of evidence for variant classification, including the published literature, population data, *in silico* prediction data, functional data, clinical phenotype and family history, and public databases (e.g., ClinVar; https://www.ncbi.nlm.nih.gov/search/all/?term=clinvar; accessed 26/08/2024).

Variants are ‘scored’ from the combined evidence into five classes:

Class 5: pathogenic.

Class 4: likely pathogenic.

Class 3: variant of uncertain significance (VUS)

Class 2: likely benign.

Class 1: benign.

Results are reported in a consistent format, using standardised nomenclature to describe the gene variant (https://hgvs-nomenclature.org/stable/; accessed 26/08/024) (Deans *et al.* 2022).

This includes the approved gene name (*RET*), reference transcript (*RET* gene NM_020975.6) and human genome build used for sequence alignment and variant assessment (GRCh37/hg19) (den Dunnen *et al.* 2016).

Currently, only pathogenic/likely pathogenic variants are routinely reported.

Reporting of VUS is variable, with the approach differing between accredited laboratories. As *RET* is an autosomal dominant gene, most laboratories will report VUS in diagnostic tests for both oncology and non-oncology patients. However, in the UK, there is a more cautious approach. VUS are considered for reporting only where there is a high level of supporting evidence and if additional evidence might be obtained to allow reclassification as (likely) pathogenic (Loong *et al.* 2022).

### Genomic test outcomes

The following outcomes are possible:

1) Confirmation of a genetic diagnosis: a *RET* pathogenic/likely pathogenic variant is identified, consistent with the patient’s condition, having been specifically solicited.

2) Incidental findings: a pathogenic/likely pathogenic *RET* variant is identified unrelated to the reason for requesting the test (e.g. after WGS analysis in a child with congenital malformation/dysmorphism). This is due to recommendations that common cancer-susceptibility genes should be assessed if/when the methodology allows, even if irrelevant to the clinical presentation (Miller *et al.* 2023).

3) Identification of a *RET* variant of uncertain significance (VUS).

4) No significant findings.

## Clinical outcomes: how to action the test result

### Care of the individual

All test outcomes should be discussed with the patient.

#### Confirmed genetic diagnosis, with a pathogenic/likely pathogenic variant

Onward referral to clinical genetics is advised to support individuals and their families in dealing with this new information (Izatt *et al.* 2022).

Management of pre-symptomatic MEN2 cases is based on the risk stratification of their genotype, as per ATA guidelines (Wells *et al.* 2015). These findings inform the timing of thyroidectomy and surveillance for phaeochromocytoma and hyperparathyroidism (Fig. 2, Table 3).

**Table 3:** The management of MEN2 patients according to the revised ATA guidelines for MTC (2015).

ATA risk category (*RET* variants)*	Timing of genomic testing	Timing of thyroidectomy	Incidence of phaeochromocytoma (PCC)	Incidence of primary hyperparathyroidism (HPTH)	Age to start screening for PCC/HPTH^†^
**Highest** M918T	At birth-cord blood Ages 0–1	Before age 1	50%	Not applicable	11 years Annual plasma metanephrines, calcium and parathyroid hormone (PTH)
**High** C634S/G/R/Y/F/W A883F	Ages 3–5	Before age 5 Screen with annual calcitonin, thyroid ultrasound and neck examination from 3 years	50%	20–30%	11 years Annual plasma metanephrines, calcium and parathyroid hormone (PTH)
**Moderate** G533C C609R/Y/F/S/W C611S/G/R/Y/F/W C618S/G/R/Y/F C620S/G/R/Y/F/W C630S/R/Y K666E/N E768D L790F V804L/M S891A S904F R912P	Age 5	From 5 years Screen with annual calcitonin, thyroid ultrasound and neck examination from 5 years. Thyroidectomy is recommended once serum calcitonin becomes elevated in childhood aged 5–10 years or adulthood	10–20%	10%	16 years Annual plasma metanephrines, calcium and parathyroid hormone (PTH)

The management of MEN2 patients according to the risk levels for the age of onset/aggressiveness of MTC, as recommended in the Revised American Thyroid Association guidelines (Wells *et al.* 2015). If genomic testing confirms hereditary MEN2, then early thyroidectomy is recommended. MEN2 care should be led by a specialist multidisciplinary team. The incidence of phaeochromocytoma and primary hyperparathyroidism is lower than that of MTC across all genotypes.

* Germline *RET* variants are listed by affected codon and the predicted protein change, using the NM_020975.6 reference sequence.

^†^ Radiological surveillance is not routinely performed in the absence of symptoms or abnormal biochemical results suggestive of PCC.

However, risk management of MEN2 cases with moderate-risk MTC variants, when ascertained as incidental findings or via population screening, may require adjustment. The natural history of disease is not fully known outside of familial cases, and MTC penetrance may be reduced. Therefore, the appropriateness of preventative thyroidectomy, as reported for *RET* V804M, the most frequent moderate-risk variant, is unclear (Loveday *et al.* 2018).

#### Uncertain results

Variants of uncertain significance need careful consideration.

The laboratory report details investigations that could provide additional evidence to facilitate variant reclassification (e.g., further investigations in the patient, their family or additional laboratory studies) (Elisei *et al.* 2012). Clinical genetics can facilitate this process (e.g., assessing segregation in other affected family members) (Newey 2022). Submission of additional information to the genomic laboratory may provide sufficient evidence to reclassify the variant upwards to a (likely) pathogenic variant. An updated report is issued by the laboratory, confirming the genetic diagnosis, to enable MEN2-focused care.

However, variant reclassification is not always possible. Clinical management (including cascade testing of unaffected family members) should not be predicated on VUS, as variants may ultimately prove to be benign.

It is not standard practice for laboratories to systematically review previously classified variants. Variant reinterpretation is typically reactive (Loong *et al.* 2022). The clinician may consider asking the laboratory to reassess the variant in 3–5 years, or earlier if new information/evidence emerges (e.g., additional affected family members/new published knowledge). If variant reinterpretation leads to upward reclassification, the result is communicated nationally between laboratories for all cases to be notified (https://www.acgs.uk.com/media/12533/uk-practice-guidelines-for-variant-classification-v12-2024.pdf; accessed 26/08/2024). Reissued reports are relayed to clinicians to action, proactively re-contacting historic patients to manage them as MEN2.

### Care of the family

#### Cascade testing

First-degree relatives should be offered a variant-specific gene test to determine their own genetic risk. If the *RET* variant is not detected, the individual can be reassured that they are not at risk of MEN2. They remain at population risk of MTC.

#### Reproductive options

Couples may want to explore options for having a child without inherited *RET* disease. These include adoption, gamete donation or not having children at all, as well as non-invasive and invasive testing.

Preimplantation genetic diagnosis uses IVF technology to create embryos that are screened for MEN2A/MEN2B or Hirschsprung disease. Only unaffected embryos are transferred to the mother. NHS funding is available for couples meeting certain eligibility criteria (e.g., the couple has no unaffected children or the female partner is <40 at the start of treatment), or couples may self-fund.

Non-invasive prenatal diagnosis (NIPD) is possible in couples where the father carries the *RET* variant, after developing a bespoke assay. NIPD tests for the paternal *RET* variant amplified from free foetal DNA, detected from 9 weeks in the mother’s blood (Macher *et al.* 2012).

Invasive prenatal diagnosis tests for the *RET* variant in a chorionic villus sample taken from the mother’s placenta from 11.5 weeks in pregnancy or in an amniotic fluid sample obtained by amniocentesis from 15 to 18 weeks in pregnancy.

Couples may decide to conceive naturally and instead seek childhood testing (including testing cord blood) to understand their child’s *RET* status.

#### Childhood testing

Childhood predictive testing for *RET* pathogenic variants is appropriate because children diagnosed with MEN2 are recommended to undergo pre-emptive thyroidectomy by 1 year (ATA highest-risk category) or by 5 years (ATA high-risk). There is variability in the age of MTC onset in moderate-risk families, but the factors that modify penetrance are not fully known (Machens & Dralle 2024). Advanced MTC in V804M from age 5 is reported (Shankar *et al.* 2021); however, if calcitonin remains normal, thyroidectomy may be delayed beyond 5 years (Fig. 2, Table 3).

## New genomic testing programmes: population screening

Genomic newborn screening (gNBS) for rare disease is being piloted globally, with the promise of increasing the number of treatable early conditions that could be detected and prevented (Stark & Scott 2023). Two studies, Early Check (https://earlycheck.org/assets/Uploads/Gene-Condition-Lists/14920_Early_Check_NC_Gene_List_group1v2.pdf; accessed 26/08/2024) and Baby Screen+ (https://babyscreen.mcri.edu.au/media/lh1duxez/babyscreen-gene-list-v1-108.pdf; accessed 19/05/2024), list *RET* as a target gene, with Early Check reporting only MEN2B cases. Over 90% of MEN2B cases are *de novo*, with the highest risk of developing MTC, often in infancy. The ability to measure calcitonin as a marker of MTC development serves as a confirmatory test of disease before early thyroidectomy with curative intent (Zenaty *et al.* 2009). Many gNBS programme, including the UK Newborn Genomes programme (https://www.genomicsengland.co.uk/initiatives/newborns; accessed 26/08/2024), are more cautious in their approach, choosing not to include *RET* as a target gene in their gNBS condition list due to uncertainties in MTC/MEN2 penetrance when ascertained at the population level. gNBS programmes continue to review and update target gene lists, and therefore, inclusion of specific *RET* variants may change as evidence emerges (https://www.genomicsengland.co.uk/initiatives/newborns/choosing-conditions; accessed 12/01/2025). Reporting the outcomes from these studies to understand the costs and long-term ethical, legal and psychosocial challenges will be fundamental to demonstrating lessons from this new approach.

## *RET*-altered cancers

### Activating *RET* mutations

#### Medullary thyroid cancer

Somatic *RET* point mutations, small deletions and/or insertions are identified in ∼55% of sporadic MTCs, with M918T being the most frequent oncogenic driver (Table 4) (Ciampi *et al.* 2019). Patients with somatic *RET* variants have more severe MTC, with an increased risk of lymph node and distant metastasis compared to those with wild-type *RET* (Salvatore *et al.* 2021).

**Table 4:** Somatic *RET* mutations in sporadic MTC.

*RET* mutation	Frequency among somatic *RET* mutations in sporadic MTC (%, *n* = 1,193)*
M918T	68.2
C634R	5
A883F	2.2
C634W	1.9
C634Y	1.9
D898_E901del	1.9
C630R	1.5
E632_L633del	1.4
C620R	0.9
S891A	0.7
C618S	0.7
C634S	0.6
C618R	0.5
Other	12.6

* Somatic *RET* mutation frequencies are derived from the COSMIC database (https://cancer.sanger.ac.uk/cosmic; COSMIC v99, released 28-NOV-23; accessed 19/05/2024).

A total of 1,193 medullary thyroid cancer samples with identified *RET* mutations are listed in the COSMIC database. The top 13 mutations are listed, with M918T being the most common. Somatic variants cluster in specific exons, as seen in germline MEN2 cases, exons 10, 11 and 13–16. Mutation frequencies less than 0.5% are not listed individually.

#### Phaeochromocytoma

Somatic point *RET* mutations are identified in ∼7% sporadic phaeochromocytomas (intra-adrenal paragangliomas) (https://cancer.sanger.ac.uk/cosmic/gene/analysis?ln=RET; accessed 19/05/2024)

### *RET* rearrangements

**Figure 3:**
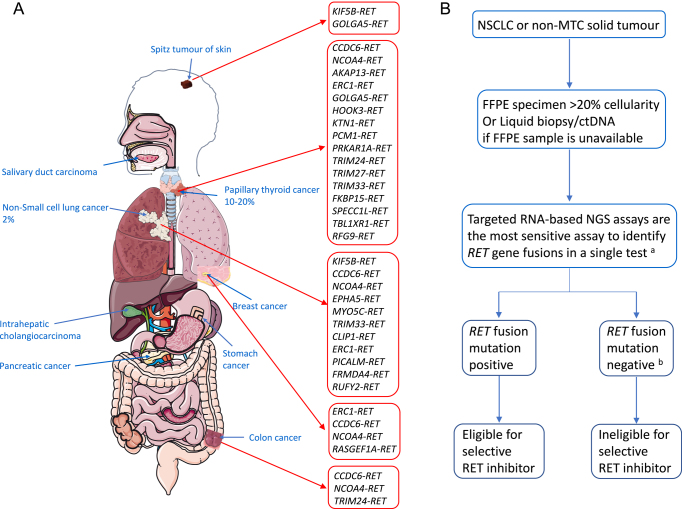
Schematic representation of *RET* fusions and partner genes at different solid tumour sites and recommended *RET* somatic testing algorithm for non-MTC solid tumours. (A) Schematic representation of somatic *RET* fusions and partner genes at different solid tumour sites. Frequencies of *RET* fusions are listed for PTC (10–20%) and non-small cell lung cancer (2%) (NSCLC), but in all other solid tumours, the frequency is very low (0–1%). *RET* gene breakpoints are mainly clustered in intron 11, with introns 7, 10 and exon 11 also observed. RET receptor activation occurs by aberrant expression or ligand-independent dimerisation with activation of RET. Red arrows indicate somatic *RET* fusions at that cancer site: skin (Spitz tumours), thyroid (papillary), NSCLC, breast and colon cancer. *CCDC6*, *NCOA4* and *KIFB* are the most common fusion partners. *KIFB* is the most frequent fusion partner in NSCLC (83.6%) and *CCDC6* in PTC (59%). Data from the COSMIC database (https://cancer.sanger.ac.uk/cosmic; COSMIC v99, released 28-NOV-23; accessed 19/05/2024). (B) Recommended *RET* somatic testing algorithm for NSCLC and non-MTC solid tumours. FFPE = formaldehyde-fixed-paraffin-embedded tissue. If a tissue sample is unavailable or exhausted, or if sufficient quality RNA cannot be extracted from an old sample, then a liquid biopsy can be performed to analyse circulating tumour DNA (ctDNA). NGS = next-generation sequencing. Data from Belli *et al.* (2021). ^a^If NGS is unavailable, FISH (fluorescence i*n *s*itu* hybridisation) or RT-PCR might also yield results, but they are less sensitive. ^b^If no *RET* fusion is detected on a liquid biopsy, then a new tissue biopsy is required to re-test for *RET* fusions to establish whether a *RET* fusion is present or not.

*RET* can combine with multiple other gene partners to create a somatically acquired oncogenic fusion protein. Over 61 different *RET* fusion genes have been reported, usually mutually exclusive to other oncogenic drivers (Parimi *et al.* 2023). *RET* chromosomal breakpoints are commonly in introns 7, 10 or 11. The chimeric protein created from *RET* breakpoints retains the intracellular kinase domain. The partner genes contribute protein dimerisation domains, which constitutively activate RET signalling via multiple mechanisms (Salvatore *et al.* 2021). The most common *RET* fusion genes in solid tumours are shown in Fig. 3.

#### Non-medullary thyroid cancer

*RET* fusions occur in 10–20% PTC cases, most commonly with *CCDC6* or *NCOA4* fusion partners (>90%) (Romei & Elisei 2012). *RET* fusions are more common in children with PTC, particularly after radiation exposure (Salvatore *et al.* 2021). The presence of *RET* fusions in PTC cases is associated with more aggressive tumour behaviour, with high rates of local lymph node and distant metastasis (Bulanova Pekova *et al.* 2023). *RET* fusions also occur infrequently in follicular, poorly differentiated, Hurtle cell or anaplastic TC. The identification of *RET* fusions in patients with advanced radioiodine-refractory TC is key to patient eligibility for targeted therapies and trials.

#### Lung cancer

*RET* fusions are found in 1–2% of NSCLC, with the *KIF5B* fusion partner found most frequently (Takeuchi *et al.* 2012).

#### Other solid tumours

*RET* fusions are detected at low frequency (0.05–1%) in multiple solid tumours (Fig. 3) (Desilets *et al.* 2023).

## Somatic testing in cancers

### Who to test

Tumour testing should be discussed with the patient, as genomic results may alter their clinical care (e.g., personalised cancer treatment). Informed consent should be sought for broad-based genomic testing, as potential pathogenic germline variants could be identified (Casolino *et al.* 2024).

### Choosing the correct solid tumour test

The NGTD for cancer lists genomic tests commissioned for oncology management and test eligibility (https://www.england.nhs.uk/publication/national-genomic-test-directories/; version 9; accessed 26/08/2024); and iterates which individuals might benefit from testing after biopsy or surgical resection (e.g., patients with particular tumour types or with advanced/metastatic cancer) (see Supplementary Table 5 (see section on Supplementary materials given at the end of the article)). Molecular assessment is not usually required in well-differentiated TCs, as the treatment plan is based on histopathology results. However, in poorly differentiated TC, anaplastic TC and MTC, molecular testing is recommended to identify oncogenic drivers that can be targeted therapeutically, as discussed in these UK pathology guidelines (https://www.rcpath.org/static/920f6f31-f2ed-445c-a523e71c2002ff7f/67693af7-0b91-4a0a-b2a8c94150a830bd/G098-Dataset-for-the-histopathological-reporting-of-thyroid-cancerfor-publication.pdf; accessed 12/01/2025).

### Testing approach for somatic *RET* alterations

*RET* alterations are best identified using massively parallel DNA and RNA sequencing from tumour samples, which can detect *RET* small variants or rearrangements in a histotype-agnostic manner (Belli *et al.* 2021). Currently, tumour-only sequencing is the standard approach, using a multigene panel to analyse multiple somatic drivers, including *RET*. As successful genomic analysis requires a minimum tumour cellularity of >20%, samples require pathology assessment before submission for testing. However, RNA assays, which detect *RET* rearrangements, may be limited by RNA quality. Analysis of circulating tumour DNA offers an alternative approach (Figs 2 and 3) (Rich *et al.* 2019).

Paired somatic-germline testing is currently reserved for specific indications, e.g., paediatric cancer WGS. WGS optimally requires fresh–frozen tissue (current NGTD requirements) and a paired blood sample for parallel analysis. WGS results take longer; therefore, in many cases, NGS-panel tumour testing may provide more timely results.

### Somatic analysis and reporting

Somatic variant classification is a two-step process, determining both pathogenicity and actionability for each variant. Identified somatic variants are annotated as described in the ‘Genomic analysis and reporting’ section and are given an oncogenicity classification (Horak *et al.* 2022).

The clinical significance of the variant is assessed for actionability using established guidelines, AMP/ASCO/CAP joint consensus or ESCAT (https://www.esmo.org/scales-and-tools/esmo-scale-for-clinical-actionability-of-molecular-targets-escat; accessed 26/08/2024) (Li *et al.* 2017). The AMP guidelines categorise somatic variants as follows:

Tier I: variants of strong clinical significance.

Tier II: variants of potential significance.

Tier III: variants of unknown significance.

Tier IV: variants of known insignificance (benign/likely benign).

The report details the actionability of identified variants and their impact on clinical care regarding diagnostic, prognostic or therapeutic implications.

### Somatic test outcomes

The following outcomes are possible:

1) Clinically significant actionable somatic variant/s detected (Tiers I and II). Variants of strong and potential clinical significance are reported along with their therapeutic implication. Standard-of-care treatments are recommended based on therapies approved by the EMA (European Medicines Agency), FDA (Food and Drug Administration) or NICE (National Institute for Health and Care Excellence). If an oncogenic *RET* mutation in MTC or *RET* fusion in a solid tumour is detected, the patient may benefit from targeted tyrosine kinase inhibitor therapy.

2) Recommendation for germline testing. A potential *RET* germline pathogenic variant might be present if insertions/deletions <50 bp >20%, or if a single nucleotide variant >30% variant allele frequency is detected, regardless of tumour type (Kuzbari *et al.* 2023).

3) Complex, or uncertain results (Tier III). Discussion at a Molecular Tumour Board is advised before the final report is issued (Berner *et al.* 2019).

4) Information on suboptimal results, where testing has failed or is incomplete. Recommendations for repeat testing on new samples are given.

5) No clinically significant findings. Further investigations are listed for consideration.

## How to action the somatic test result

### Post-test management

#### Clinically actionable *RET* somatic variant

The clinician discusses the result at a multidisciplinary meeting to consider the therapeutic implications of the recommended targeted therapy relative to other treatments, and a precision oncological plan is devised. The options are discussed with the patient, and treatment is commenced (Filetti *et al.* 2022).

The RET-selective inhibitors selpercatinib or pralsetinib are the current targeted therapeutic choices, as they have greater efficacy and fewer adverse events compared to multikinase inhibitors (e.g., vandetanib) (Novello *et al.* 2023). In addition, selpercatinib has been shown to have greater efficacy in phase III trials over standard-of-care therapy, with an overall response rate of 69.4 versus 38.8% in controls for patients with *RET*-positive MTC (Hadoux *et al.* 2023).

Selpercatinib is approved for use in Europe, the US and the UK for *RET*-altered TC and NSCLC. Pralsetinib is approved in the US and Europe for specific indications but not in the UK; however, this is a constantly evolving landscape.

#### Potential germline pathogenic variants

A variant found on tumour testing may be somatic (i.e., only present in the tumour tissue) or germline (i.e., present in every cell of an individual, including their tumour cells). Thus, if a potential *RET* pathogenic variant is identified, confirmatory germline testing is recommended (Kuzbari *et al.* 2023). This involves testing a blood or saliva sample for the specific *RET* variant, noting that this will require specific consent.

If a germline MEN2 *RET* pathogenic variant is confirmed, management is as previously described (see the ‘Care of the individual’ section above), in addition to treatment of the primary tumour.

## Conclusions

Genomic testing has revolutionised care of individuals with *RET*-associated disease – confirming diagnoses to guide disease management, pre-emptive thyroidectomy and personalised care. Families with inherited *RET* disease are supported by genetic teams to access predictive and childhood testing and consider reproductive options.

However, the majority of MTC cases are sporadic, and 35% present with advanced disease, with poorer outcomes compared to hereditary disease (Saltiki *et al.* 2019).

The development of RET-selective inhibitors provides an opportunity for precision therapy in those proven to have *RET*-driven tumours, particularly MTC, PTC and NSCLC. Timely and accurate cancer genomic profiling to understand oncogenic drivers is paramount, but implementation needs to be resourced equitably for patient benefit, and barriers in implementation exist (Segall *et al.* 2024, Taniere *et al.* 2024).

NGS has transformed diagnostic genomic testing in the last decade. In the future, long-read sequencing technology and integrating multimodal data are expected to accelerate precision oncology and rare disease discovery. Adoption of these new technologies holds much promise if they can become part of routine care.

## Supplementary materials



## Declaration of interest

The author declares that there is no conflict of interest that could be perceived as prejudicing the impartiality of this review.

## Funding

This work did not receive any specific grant from any agency in the public, commercial or not-for-profit sector.
