# Supplementary material for: Genomic testing for RET in the clinic: UK and global perspective
Source: Endocr Relat Cancer. 2025 Apr 15;32(5):e240230. doi: 10.1530/ERC-24-0230 (PMC12020483; doi:10.1530/ERC-24-0230)
Supplement: Supplementary file 1 [file supplementary_materials.pdf]

Supplementary Table 5. Current DNA and RNA Solid tumour NGS panel tests in non-small cell lung cancer, thyroid cancer and phaeochromocytoma

Clinical Indication	Test name	Target Genes	Test scope	Technology
Non-Small Cell Lung cancer	Multi-target NGS panel – small variant *(EGFR, ALK, BRAF, KRAS, MET, ERBB2)*	*EGFR, ALK, BRAF, KRAS* p.(G12C*), MET* exon 14 skipping, ERBB2 exon 20 insertions, ERBB2 amplifications	Small variant detection (DNA)	Panel
Non-Small Cell Lung cancer	Multi-target NGS panel - structural variant *(RET, ROS1, EML4-ALK, NTRK1, NTRK2, NTRK3, MET)*	*RET, ROS1, EML4-ALK, NTRK1, NTRK2, NTRK3, MET 14* exon skipping	Structural variant detection (RNA)	Panel
Thyroid Papillary carcinoma	Multi-target NGS panel - small variant (*BRAF, KRAS, NRAS, HRAS, TERT* promoter, *MET, ERBB2*)	*BRAF, KRAS, NRAS, HRAS, TERT* promoter, *MET* exon 14 skipping, ERBB2 exon 20 insertions, ERBB2 amplifications	Small variant detection (DNA)	Panel
Thyroid Papillary carcinoma	Multi-target NGS panel - structural variant *(ALK, MET, ROS1, RET, NTRK1, NTRK2, NTRK3)*	*ALK, MET exon 14 skipping, ROS1, RET, NTRK1, NTRK2, NTRK3*	Structural variant detection (RNA)	Panel
Thyroid Follicular carcinoma	Multi-target NGS panel - small variant (*KRAS, NRAS, HRAS, BRAF, MET, ERBB2*)	*KRAS, NRAS, HRAS, BRAF V600*, MET exon 14 skipping, MET amplifications, ERBB2 exon 20 insertions, ERBB2 amplifications*	Small variant detection (DNA)	Panel
Thyroid Follicular carcinoma	Multi-target NGS panel - structural variant *(RET, NTRK1, NTRK2, NTRK3)*	*RET, NTRK1, NTRK2, NTRK3*	Structural variant detection (RNA)	Panel
Poorly differentiated Anaplastic Thyroid carcinoma	Multi-target NGS panel - small variant *(TP53, BRAF, MET, ERBB2)*	*TP53, BRAF, MET exon 14 skipping, MET amplifications, ERBB2 exon 20 insertions, ERBB2 amplifications*	Small variant detection (DNA)	Panel
Poorly differentiated Anaplastic Thyroid carcinoma	Multi-target NGS panel - copy number variant *(TP53)*	*TP53*	Copy number variant detection to exon level resolution (DNA)	Panel
Poorly differentiated Anaplastic Thyroid carcinoma	Multi-target NGS panel - structural variant (*RET*, *NTRK1, NTRK2, NTRK3, ALK, MET, ROS1*)	*RET, NTRK1, NTRK2, NTRK3, ALK, MET exon 14 skipping, ROS1*	Structural variant detection (RNA)	Panel
Thyroid Hurtle cell carcinoma	Multi-target NGS panel - structural variant (*RET, ALK, MET, ROS1*)	*RET, ALK, MET, exon 14 skipping, ROS1*	Structural variant detection (RNA)	Panel
Medullary thyroid cancer	Multi-target NGS panel - small variant (*RET, BRAF, MET, ERBB2*)	*RET, BRAF600*, MET exon 14 skipping, MET amplifications, ERBB2 exon 20 insertions, ERBB2*	Small variant detection (DNA)	Panel

		amplifications		
Medullary thyroid cancer	Multi-target NGS panel - structural variant *(NTRK1, NTRK2, NTRK3, ALK, MET, ROS1)*	*NTRK1, NTRK2, NTRK3, ALK, MET exon 14 skipping, ROS1*	Structural variant detection (RNA)	Panel
Phaeochromocytoma	Multi-target NGS panel - small variant (*RET, BRAF, MET, ERBB2*)	*RET, BRAF600*, MET exon 14 skipping, MET amplifications, ERBB2 exon 20 insertions, ERBB2 amplifications*	Small variant detection (DNA)	Panel
Phaeochromocytoma	Multi-target NGS panel - structural variant (*NTRK1, NTRK2, NTRK3, ALK, MET, ROS1)*	*NTRK1, NTRK2, NTRK3, ALK, MET exon 14 skipping, ROS1*	Structural variant detection (RNA)	Panel

Subtypes of thyroid cancer are categorised through cytology and/or histopathology. *RET* analysis is undertaken alongside other recommended genes for that tumour type, to provide diagnostic, prognostic and therapeutic information. Gene fusions are detected on RNA analysis and small variants on DNA analysis by next-generation sequencing. Any somatic variants detected are assessed for both oncogenicity and clinical actionability to provide diagnostic, prognostic and therapeutic information for precision cancer care. Panels are listed in the National Genomes Test Directory for Cancer (https://www.england.nhs.uk/publication/national-genomic-test-directories/ version 9 accessed: 26082024)
